# Impact of COVID‐19 on the distribution of pathogenic bacteria in the lower respiratory tract of the elderly

**DOI:** 10.1002/iid3.931

**Published:** 2023-07-12

**Authors:** Shi‐Yan Zhang, Jing Shi, Ying Zhuo, Ting‐Qiang Wang

**Affiliations:** ^1^ Department of Clinical Laboratory, Fuding Hospital Fujian University of Traditional Chinese Medicine Fuding Fujian China

**Keywords:** *acinetobacter baumannii*, bacterial infection, COVID‐19, elderly, lower respiratory tract, *stenotrophomonas maltophilia*

## Abstract

**Background:**

To investigate the distribution of bacterial pathogens of lower respiratory tract infection (LRTI) in hospitalized elderly patients during the COVID‐19 epidemic and to explore the influence of COVID‐19 on the distribution of bacterial pathogens, to provide guidance for clinical diagnosis.

**Methods:**

Specimens of sputum from elderly LRTIs patients at Fuding Hospital of China were consecutively collected from October 2022 to January 2023. Cultures and identification were done, and RT‐PCR was employed to detect SARS‐Cov‐2 nucleic acid.

**Results:**

A total of 195 isolates were characterized in 163 sputum samples of consecutive hospitalized elderly patients, of which 11.3% were Gram‐positive bacteria and 88.7% were Gram‐negative. The top of frequently isolated pathogens was *Klebsiella pneumonia* (30.3%), *Pseudomonas aeruginosa* (19.0%), *Acinetobacter baumannii* (12.8%), *Stenotrophomonas maltophili*, (7.7%), *Escherichia coli* (7.2%). According to the results of novel coronavirus nucleic acid detection, the 163 patients were divided into COVID‐19 group and non‐COVID control (CNT) group. The comparison of bacterial distribution between the groups revealed that *Stenotrophomonas maltophilia* was lower in the COVID‐19 than in the CNT group, while *A. baumannii* was higher in the COVID‐19 group, and the difference was statistically significant (*p* < .05).

**Conclusion:**

The major bacteria identified in sputum culture of hospitalized elderly patients were *K. pneumonia*, *P. aeruginosa*, *A. baumannii*, *S. maltophilia*, and *E. coli*. Furthermore, the distribution of *S. maltophilia* and *A. baumannii* between the COVID‐19 and CNT groups was found to be significantly different (*p* < .05), while there were no significant differences in the distribution of other bacteria.

## Graphical abstract

The major bacteria identified in sputum culture of hospitalized elderly patients were *Klebsiella pneumonia, Pseudomonas aeruginosa, Acinetobacter baumannii, Stenotrophomonas maltophilia*, and *Escherichia coli*. The distribution of *Stenotrophomonas maltophilia* and Acinetobacter baumannii between the COVID‐19 and CNT groups was found to be significantly different (*p* < .05).

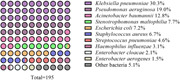

## 1 INTRODUCTION

Lower respiratory tract infection (LRTI) is one of the common infectious diseases among the elderly, and with the pandemic of the century, the combination of the two will pose a serious threat to the health of the elderly. Therefore, the study of the bacterial distribution indexes between LRTI and novel coronavirus infection in elderly people over 60 years old is helpful for medical workers to better identify and judge in clinical practice.^1^

Age has been identified as a major factor influencing the outcomes of COVID‐19. Elderly individuals (over the age of 60) are more likely to be admitted to the intensive care unit and have a higher mortality rate due to their immunosenescence and the presence of multiple comorbidities.^2^, ^3^ The risk factors for LRTIs are also not nearly the same at different ages, and older people are potentially more at risk than younger people and less able to recover from the disease. This is also the case with COVID‐19, with the elderly also more likely to have both COVID‐19 and LRTIs than other groups.^4^ To better understand the prevalence of LRTIs in elderly patients, sputum samples from inpatients aged over 60 were collected and subjected to culturing.

LRTIs are common bacterial infections among hospitalized patients, with a mortality rate of up to 3%–5% in adults, particularly the elderly,^5^, ^6^ these infections can be further complicated by the emergence of the novel coronavirus. Elderly patients with multiple underlying diseases and decreased immune function are more vulnerable to the new coronavirus infection, which is often accompanied by lower respiratory tract bacterial infection.

The incidence, prevalence, and characteristics of LRTIs in elderly COVID‐19 patients is not well known and has been raised as a vital knowledge gap.^7^ However, a comparison of relative frequency and microbiological characteristics of the pathogenic distribution in LRTIs admitted to hospitalizations had not been previously investigated in China. The aim of this study was to provide valuable insights into the prevalence and distribution of LRTI bacterial pathogens in elderly patients while also examining the complex interaction between these pathogens and the novel coronavirus. Additionally, we explored the various pathogen that influenced the clinical outcomes of elderly LRTI patients during the COVID‐19 pandemic. The knowledge gained from our research would be instrumental in informing clinical decision‐making and developing tailored treatment approaches for elderly LRTI patients, ultimately improving patient care and outcomes in this vulnerable population.

## 2 MATERIALS AND METHODS

This retrospective study included 163 elderly LRTI consecutive patients admitted to the hospital between October 2022 and January 2023, aged 60–95 years old (see Table 1 for more details). All patients underwent routine bacterial culture, isolation, and identification of pathogenic bacteria from sputum specimens, and RT‐PCR method was used to determine the coronavirus nucleic acid. This study was approved by the Medical Ethics Committee of Fuding Hospital (The ethnical approval number: Fuding 2023001).

**Table 1:** Clinical parameters of COVID‐19 group and CNT group [case (%)].

Items	CNT (*n* = 79)	COVID‐19 (*n* = 84)	Mann–Whitney *U*/*χ* ^2^ test	*p* value
Age (years) (range)	(61–95）	(60–94)		
Age median (P_25_–P_75_)	71.0 (66.0–79.0)	73.5 (67.0–79.0)	−0.723	.470
Gender			1.800	.180
Male	67 (57.9)	62 (56.3)		
Female	12 (42.1)	22 (43.7)		

*Note*: Kolmogorov–Sminov test was carried out in the age of COVID‐19 group and CNT group: COVID‐19 (*Z* = 0.086, *p* = .162), CNT (*Z* = 0.162, *p* < .001). CNT: non‐COVID control subjects.

Bacterial isolation and identification were conducted according to the third edition of the National Guide for Clinical Laboratory Procedures. Bacterial analyzers and identification cards were used for the isolation and identification of pathogens. Before the patient received anti‐infective therapy upon admission, a nasopharyngeal swab was tested for coronavirus nucleic acid, and sputum, suction sputum, tracheal secretions, and deep tracheal secretions were also obtained and immediately sent to the laboratory for bacterial culture. The laboratory conducted a preliminary screening by microscopy, Gram staining and low‐power observation of single‐field squamous epithelial cells less than 10 and more than 25 neutrophils were considered qualified sputum specimens. Qualified sputum specimens were inoculated on Blood agar plates and cultured in a 35°C incubator for 24–48 h to isolate and purify the dominant bacteria and make an identification. Microorganisms were identified performing a VITEK 2 identification system.

The control strains used: *Staphylococcus aureus* ATCC 25923, *Escherichia coli* ATCC 25922, *Enterococcus faecalis* ATCC 29212, and *Pseudomonas aeruginosa* ATCC 27853 were all obtained from the Fujian Provincial Inspection Center. The coronavirus nucleic acid test was performed by RT‐PCR, and the reagents, positive and negative control reagents, automatic nucleic acid extractor, and fluorescent PCR instrument were all provided by Xi'an Tianlong Gene Co., Ltd. Ct values (cycle threshold values) less than 40 were judged as positive results.

Data processing was performed using the SPSS version 22.0 software (SPSS Inc.), and the count data was expressed as percentages. The Chi‐square test (*χ* ^2^) or Fisher's exact test was used for intergroup comparison. Fisher's exact test was applied as numbers in one or more categories were <5. Testing of data for normal distribution was performed by means of Kolmogorov–Smirmov test. For metric data that had passed the normality test, the median and quartiles (P_25_–P_75_) were used to represent the data. Mann–Whitney *U* test (nonparametric test) was used for data comparison. In all tests, a *p* < .05 was considered statistically significant.

## 3 RESULTS

A total of 163 hospitalized elderly patients, including 129 males and 34 females, were included in this study, with 84 patients in the COVID‐19 group and 79 in the CNT group. The age of the patients in the two groups was tested by Kolmogorov–Sminov test, with the results of COVID‐19 group *Z* = 0.086, *p* = .162 and CNT group *Z* = 0.162, *p* < .001. The data showed a non‐normal distribution, and statistical analysis was performed by Mann–Whitney *U* test (nonparametric test). Chi‐square test was used to compare the gender between the two groups. The test results of age and gender were shown in Table 1, and there was no statistically significant difference (*p* > .05).

**Figure 1:**
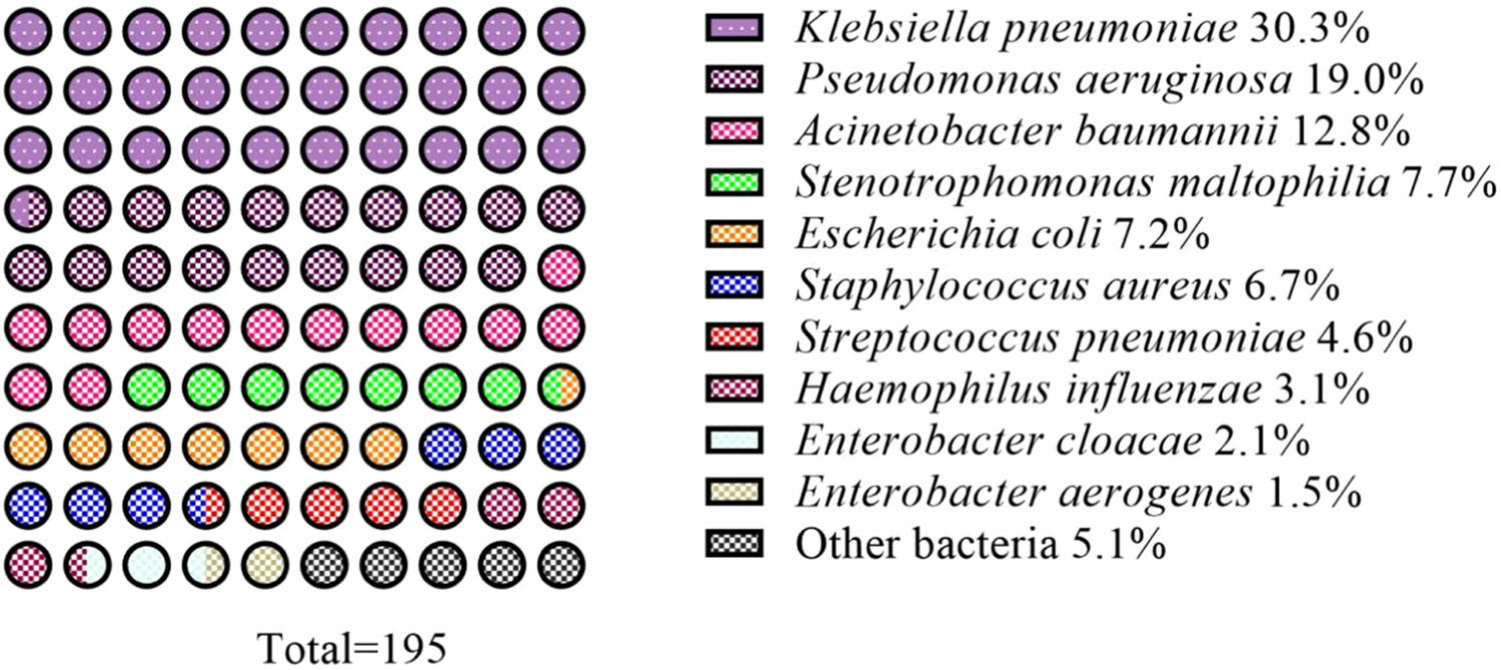
The distribution of the top of 10 frequently isolated pathogens.

Among 163 hospitalized elderly patients with sputum samples, 195 strains of bacteria were isolated, including 22 Gram‐positive bacteria (11.3%) and 178 Gram‐negative bacteria (88.7%). The top 10 pathogenic bacteria were *Klebsiella pneumonia, P. aeruginosa, Acinetobacter baumannii, Stenotrophomonas maltophilia, E. coli, S. aureus, Streptococcus pneumoniae, Haemophilus influenza, Enterobacter cloacae*, and *Enterobacter aerogenes*. accounting for 30.3%, 19.0%, 12.8%, 7.7%, 7.2%, 6.7%, 4.6%, 3.1%, 2.1%, and 1.5%, respectively, and the remaining bacteria accounted for 5.1% (see Table 2 and Figure 1).

**Table 2:** Comparison of pathogenic bacteria between COVID‐19 group and CNT group [*n* (%)].

Bacteria	CNT (*n* = 97)		COVID‐19 (*n* = 98)	*χ* ^2^	*p* value	Two groups (*n* = 195)
**Gram‐positive bacterium**		12 (12.4)	10 (10.2)			22 (11.3)
*S. pneumoniae*	Yes	4 (4.1)	5 (5.1)	0.105	.745	9 (4.6)
	No	93 (95.9)	93 (94.9)			
*S. aureus*	Yes	8 (8.2)	5 (5.1)	0.775	.379	13 (6.7)
	No	89 (91.8)	93 (94.9)			
**Gram‐negative bacterium**		85 (87.6)	88 (89.8)			173 (88.7)
*K. pneumoniae*	Yes	29 (29.9)	30 (30.6)	0.012	.913	59 (30.3)
	No	68 (70.1)	68 (69.4)			
*P. aeruginosa*	Yes	15 (15.5)	22 (22.4)	1.547	.214	37 (19.0)
	No	82 (84.5)	76 (77.6)			
A. *baumannii*	Yes	18 (18.6)	7 (7.1)	5.682	.017	25 (12.8)
	No	79 (81.4)	91 (92.9)			
*S. maltophilia*	Yes	3 (3.1)	12 (12.2)	5.751	.016	15 (7.7)
	No	94 (96.9)	86 (87.8)			
*E. coli*	Yes	10 (10.3)	4 (4.1)	2.823	.093	14 (7.2)
	No	87 (89.7)	94 (95.9)			
*H. influenzae*	Yes	2 (2.1)	4 (4.1)	0.663	.415	6 (3.1)
	No	95 (97.9)	94 (95.9)			
*E. cloacae*	Yes	3 (3.1)	1 (1.0)	1.037	.309	4 (2.1)
	No	94 (96.9)	97 (99.0)			
*E. aerogenes*	Yes	1 (1.0)	2 (2.0)	0.327	.568	3 (1.5)
	No	96 (99.0)	96 (98.0)			
*Other bacteria*		4	6			10 (5.1)

*Note*: CNT: non‐COVID control subjects. Other bacteria: *L. adecarboxylate* (1 case), *R. ornithinolytica* (1), *R. pickettii* (1), *K. oxytoca* (1) in the CNT group; *A. denitrificans* (1), *M. catarrhalis* (1), *A. hydrophila* (1), *S. marcescens* (2 cases) in the COVID‐19 group.

Single bacteria infection occurred in 163 cases (82.2%, 134/163) of hospitalized elderly patients, and mixed infection with two bacteria occurred in 25 cases (16.0%, 26/163), and mixed infection with three bacteria occurred in 3 cases (1.8%, 3/163).

The distribution of bacteria in COVID‐19 group (98 strains) and CNT group (97 strains) was analyzed. Except for *A. baumannii* and *S. maltophilia*, there was no statistical difference in the distribution of other strains. The composition ratio of *A. baumannii* in the COVID‐19 group (7.1%) was lower than that in the CNT group (18.6%), the difference was statistically significant (*p* = .017); the composition ratio of *S. maltophilia* in the COVID‐19 group (12.2%) was higher than that in the CNT group (3.1%), the difference was statistically significant (*p* = .016) (see Table 2).

## 4 DISCUSSION AND CONCLUSION

The novel coronavirus has been found to have membrane glycoprotein, spike protein, nucleocapsid protein, envelope protein and coagulase.^8^ The spike glycoprotein on the surface of the virus plays a major role in its attachment and entry into host cells, and the infection can lead to lethal damage in the lungs, heart, kidneys, circulatory system, gastrointestinal tract and nervous system tissues.^9^ Elderly people are particularly vulnerable to the virus due to their weakened immune systems, and evidence suggests that a large number of hospitalized patients during the COVID‐19 pandemic had mixed bacterial infections and secondary bacterial infections, which were a major cause of higher mortality in elderly patients.^10^ When the novel coronavirus invades the lungs, it causes damage to the lung cells and tissues, which attracts neutrophils and macrophages to the infected site and promotes inflammation, and eventually leads to bacterial adhesion and invasion into the cells and proliferation.^11^ Therefore, it is essential to identify the bacterial pathogens of LRTIs in the elderly.

The results of this study showed that *K. pneumoniae, P. aeruginosa, A. baumannii, S. maltophilia, E. coli, S. aureus*, and *S. pneumoniae* were the main pathogenic bacteria in the sputum specimens of 163 hospitalized elderly patients in this study. This is consistent with similar studies,^12^ which showed that the common bacteria identified in respiratory cultures, even the sputum culture results of elderly patients with pulmonary infections, were mostly Gram‐negative bacteria, mainly including *K. pneumoniae, E. coli, P. aeruginosa, Bacteroides fragilis*, etc.; the Gram‐positive bacteria infected included *S. pneumoniae, S. aureus*, etc. *K. pneumoniae* is a Gram‐negative bacterium with a thick capsule, mostly distributed in the human respiratory and gastrointestinal tracts, and is a common pathogen for elderly respiratory infections. Said k et al.^13^ conducted a screening for combined microbial infection in 301 COVID‐19 patients, and the main microorganisms detected were *K. pneumoniae* (37%, 48/301), *P. aeruginosa* (8.5%, 11/301), and *E. coli* (18.6%, 24/301).

The opportunistic Gram‐negative bacillus *S. maltophilia* is known to infect mostly elderly patients. According to one study, individuals over the age of 60 were more prone to *S. maltophilia* infection, which could be attributed to immunosuppression.^14^ In recent years, *S. maltophilia* has been relatively stable in third place among non‐fermenting Gram‐negative bacteria, after *P. aeruginosa* and *A. baumannii*, according to the CHINET monitoring service.^15^ Patients with severely impaired or weakened immune functions were more susceptible to serious consequences when infected with *S. maltophilia*, suggesting that the respiratory tract was the main site of colonization and infection of *S. maltophilia*, which may have been associated with its ability to form biofilms and colonize the respiratory tract of hospitalized patients.^16^ Recently, *S. maltophilia* was reported as one of the secondary bacterial infections in ICU patients with COVID‐19,^12^ which is similar to what was found in this study: the infection rate of *S. maltophilia* was higher in the COVID‐19 group than in the CNT group.

*A. baumannii* is a Gram‐negative bacterium with strong vitality, and it is a major opportunistic pathogen that can cause double infection, especially in hospitalized patients with viral respiratory infection.^17^ Recent studies have shown that the infection rate of *A. baumannii* in COVID‐19 patients varies widely in different regions. For example, Nanshan chen et al.^18^ reported that among 99 COVID‐19 patients in Wuhan, only one was cultured with *A. baumannii*, with an infection rate of 10.1% (1/99); another domestic report showed that among 1495 hospitalized COVID‐19 patients in Wuhan, 102 (6.8%) had bacterial mixed infection, mainly *A. baumannii* (35.8%), and nearly half (49.0%, 50/102) died during hospitalization^19^; Iranian scholars Sharifipour et al.^17^ reported that 17 out of 19 elderly COVID‐19 patients had *A. baumannii* infection, with an infection rate of 89.5% (17/19); Brazilian researchers Silva et al.^20^ reported that the infection rate of *A. baumannii* was 32.8% (21/64); Said et al.^13^ in Saudi Arabia reported that 34 of the 301 COVID‐19 patients had drug‐resistant *A. baumannii* (26%, *n* = 34).

The infection rate of *A. baumannii* varies in different regions, and further research is needed to investigate the specific reasons. In the hospital environment, COVID‐19 elderly LRTIs patients may face another threat to health, which is mixed bacterial infection and nosocomial secondary bacterial infection, especially the mixed infection and secondary infection of *S. aureus* and *A. baumannii*, which may seriously affect the clinical outcome of COVID‐19 patients.

Whereas, This study has certain limitations, for it was solely focused on bacteria, not including fungi, mycoplasma, chlamydia, parasites and other viruses into the study of pathogens in elderly LRTIs patients; The number of patients enrolled is small, which could result in a sample size bias, therefore, large‐scale clinical studies need to be conducted to investigate the incidence, prevalence, characteristics and microbiological distribution of COVID‐19 complicated infections. As a result, a multicenter, prospective, longitudinal study is required to confirm the research.

In conclusion, the current study has identified the main bacterial pathogens of elderly LRTIs patients and has highlighted the importance of identifying and treating secondary bacterial infections in elderly patients with COVID‐19. Furthermore, the findings of this study suggest that *K. pneumoniae, P. aeruginosa, A. baumannii, S. maltophilia, E. coli, S. aureus*, and *S. pneumoniae* are the main bacterial pathogens of elderly LRTIs patients, and that *S. maltophilia* is an opportunistic pathogen that is particularly dangerous for elderly patients with weakened immune systems.

## AUTHOR CONTRIBUTIONS

**Shi‐Yan Zhang**: conceptualization; formal analysis; software; writing—original draft; writing—review & editing. **Jing Shi**: methodology; writing—review & editing. **Ying Zhuo**: conceptualization; writing—original draft; writing—review & editing. **Ting‐Qiang Wang**: software; writing—review & editing.

## CONFLICT OF INTEREST STATEMENT

The authors declare no conflicts of interest.

## Data Availability

Research data are not shared.
